# External Validation and Extension of a Clinical Score for the Discrimination of Type 2 Myocardial Infarction

**DOI:** 10.3390/jcm10061264

**Published:** 2021-03-18

**Authors:** Thomas Nestelberger, Pedro Lopez-Ayala, Jasper Boeddinghaus, Ivo Strebel, Maria Rubini Gimenez, Iris Huber, Karin Wildi, Desiree Wussler, Luca Koechlin, Alexandra Prepoudis, Danielle M. Gualandro, Christian Puelacher, Noemi Glarner, Philip Haaf, Simon Frey, Adam Bakula, Rupprecht Wick, Òscar Miró, F. Javier Martin-Sanchez, Damian Kawecki, Dagmar Keller, Raphael Twerenbold, Christian Mueller

**Affiliations:** 1 Cardiovascular Research Institute Basel (CRIB) and Department of Cardiology, University Hospital Basel, University of Basel, 4031 Basel, Switzerland; thomas.nestelberger@usb.ch (T.N.); pedro.lopezayala@usb.ch (P.L.-A.); jasper.boeddinghaus@usb.ch (J.B.); ivo.strebel@usb.ch (I.S.); maria.rubini@usb.ch (M.R.G.); iris.huber@usb.ch (I.H.); wildik@hotmail.com (K.W.); desireenadine.wussler@usb.ch (D.W.); luca.koechlin@usb.ch (L.K.); alexandra.prepoudis@usb.ch (A.P.); danielle.gualandro@usb.ch (D.M.G.); christian.puelacher@usb.ch (C.P.); noemi.glarner@usb.ch (N.G.); philip.haaf@usb.ch (P.H.); simon.frey@usb.ch (S.F.); adam.bakula@pm.me (A.B.); Rupprecht.wick@usb.ch (R.W.); raphael.twerenbold@usb.ch (R.T.); 2 GREAT Network, 00191 Rome, Italy; omiro@clinic.cat (Ò.M.); fjjms@hotmail.com (F.J.M.-S.); d.kawecki@interia.pl (D.K.); 3 Division of Cardiology, Vancouver General Hospital, University of British Columbia, Vancouver, BC V5Z 1M9, Canada; 4 Department of Internal Medicine/Cardiology, Heart Center Leipzig at University Leipzig, 04109 Leipzig, Germany; 5 Critical Care Research Institute, the Prince Charles Hospital, Brisbane and University of Queensland, 4072 Brisbane, Australia; 6 Department of Cardiac Surgery, University Hospital Basel, 3010 Basel, Switzerland; 7 Emergency Department, Hospital Clinic, 08036 Barcelona, Spain; 8 Servicio de Urgencias, Hospital Clínico San Carlos, 28040 Madrid, Spain; 9 2nd Department of Cardiology, School of Medicine with the Division of Dentistry in Zabrze, Medical University of Silesia, 40-055 Katowice, Poland; 10 Emergency Department, University Hospital Zurich, 8006 Zurich, Switzerland; dagmar.keller@usb.ch

**Keywords:** type 1 myocardial infarction, type 2 myocardial infarction, differentiation, external validation, risk scores

## Abstract

Background: The early non-invasive discrimination of Type 2 versus Type 1 Myocardial Infarction (T2MI, T1MI) is a major unmet clinical need. We aimed to externally validate a recently derived clinical score (Neumann) combing female sex, no radiating chest pain, and high-sensitivity cardiac troponin I (hs-cTnI) concentration ≤40.8 ng/L. Methods: Patients presenting with acute chest discomfort to the emergency department were prospectively enrolled into an international multicenter diagnostic study. The final diagnoses of T2MI and T1MI were centrally adjudicated by two independent cardiologists using all information including cardiac imaging and serial measurements of hs-cTnT/I according to the fourth universal definition of MI. Model performance for T2MI diagnosis was assessed by formal tests and graphical means of discrimination and calibration. Results: Among 6684 enrolled patients, MI was the adjudicated final diagnosis in 1079 (19%) patients, of which 242 (22%) had T2MI. External validation of the Neumann Score showed a moderate discrimination (C-statistic 0.67 (95%CI 0.64–0.71)). Model calibration showed underestimation of the predicted probabilities of having T2MI for low point scores. Model extension by adding the binary variable heart rate >120/min significantly improved model performance (C-statistic 0.73 (95% CI 0.70–0.76, *p* < 0.001) and had good calibration. Patients with the highest score values of 3 (Neumann Score, 9.9%) and 5 (Extended Neumann Score, 3.3%) had a 53% and 91% predicted probability of T2MI, respectively. Conclusion: The Neumann Score provided moderate discrimination and suboptimal calibration. Extending the Neumann Score by adding heart rate >120/min improved the model’s performance.

## 1. Introduction

Myocardial infarction (MI) remains the most common cause of death worldwide [1,2]. The clinical introduction of high-sensitivity cardiac troponin (hs-cTn) assays has enabled a more accurate diagnosis of MI [1,2]. Furthermore, it has facilitated the recognition that in a relevant proportion of patients with MIs, supply–demand mismatch due to impaired systemic hemodynamics including hypotension, hypertension, tachycardia, or hypoxemia—rather than coronary atherothrombosis (Type 1 MI (T1MI))—is the underlying pathophysiology (Type 2 MI (T2MI)) [3,4,5,6,7,8,9]. As treatments differ substantially between T2MI and T1MI, [1,2] their early and accurate non-invasive differentiation is a major, yet largely unmet clinical need. When taken individually, clinical characteristics have limited diagnostic accuracy for the early non-invasive differentiation of T2MI versus T1MI. A recent single-center pilot study introduced a multivariable score (Neumann Score) that includes female sex, no radiating chest pain, and hs-cTnI ≤40.8 ng/L (Abbott Architect) for the differentiation between T2MI and T1MI in the emergency department (ED) [10].

We therefore aimed to, first, externally validate the Neumann Score for the discrimination of T2MI versus T1MI in a large international diagnostic multicenter study and, second, assess whether its performance can be improved by incorporating other routinely available clinical variables.

## 2. Methods

### 2.1. Study Design and Population

Advantageous Predictors of Acute Coronary Syndrome Evaluation (APACE) is an ongoing prospective multicenter international diagnostic study including 12 centers in 5 countries aiming to advance the early diagnosis of MI (ClinicalTrials.gov (accessed on 3 March 2021) registry, number NCT00470587) [5].

**Figure 1:**
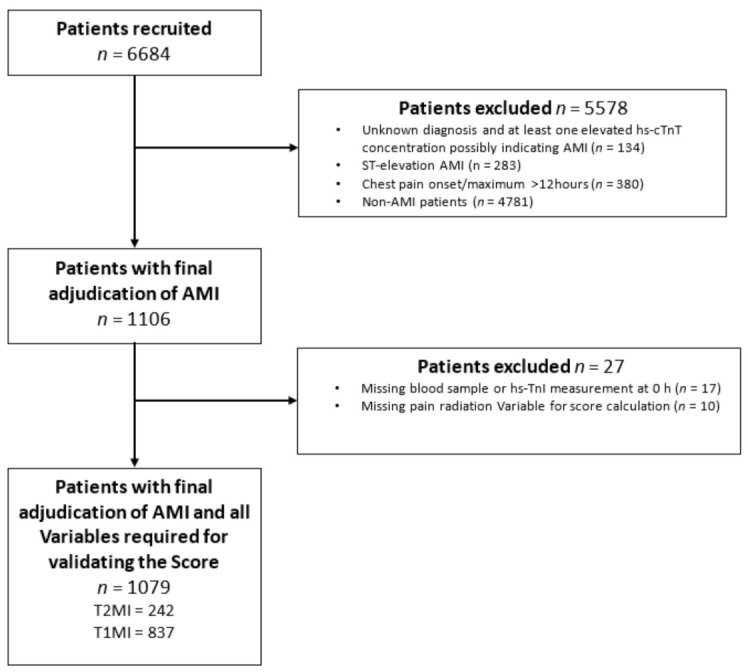
Patient flow. AMI, acute myocardial infarction, hs-cTnI, high-sensitivity cardiac Troponin I.

Adult patients presenting to the ED with symptoms suggestive of MI, such as acute chest discomfort or angina pectoris with a chest pain onset or maximum within 12 h prior to presentation, were recruited. For this analysis, patients were excluded if (A) they presented with ST-elevation myocardial infarction (STEMI), as T2MI rarely presents as STEMI, (B) the final diagnosis remained unclear even after final adjudication and had at least one elevated hs-cTn concentration, thereby possibly indicating MI, (C) patients presenting with chest pain onset/maximum >12 h, (D) terminal kidney failure requiring dialysis, (E) final adjudication was other than MI, as the Neumann Score is applied only for MI patients, and (F) a component of the Neumann Score was missing. (Figure 1).

The study was carried out according to the principles of the Declaration of Helsinki and approved by the local ethics committees. Written informed consent was obtained from all patients. The authors designed the study, gathered, and analyzed the data according to the Transparent Reporting of a multivariable prediction model for Individual Prognosis or Diagnosis (TRIPOD) statement (Supplementary Table S1) [11], vouched for the data and analysis, wrote the paper, and decided to publish. The assays were donated by the manufacturers, who had no role in the design of the study, data analysis, manuscript preparation, or decision to submit for publication.

### 2.2. Routine Clinical Assessment

All patients underwent clinical assessment that included standardized and detailed medical history, vital signs, physical examination, 12-lead electrocardiogram (ECG), continuous ECG rhythm monitoring, pulse oximetry, standard blood test, as well as non-invasive and invasive cardiac imaging as indicated. Vital signs and chest pain characteristics used for the score were assessed immediately after ED presentation. Cardiac troponin levels, including hs-cTn in some centers, were measured at presentation and serially thereafter if clinically indicated. Treatment of patients was left to the discretion of the attending physician. The estimated glomerular filtration rate (eGFR) was estimated using the chronic kidney disease epidemiology collaboration (chronic kidney disease-modification of diet in renal disease; CKD-MDRD) formula [12].

### 2.3. Central Adjudication of T1MI and T2MI

Two independent cardiologists reviewed all available medical records including patient history, physical examination, vital signs in the ambulance and in the ED, results of laboratory testing, radiologic testing, ECG, echocardiography, cardiac magnetic resonance imaging, lesion severity and morphology in coronary angiography pertaining to the patient. In situations of disagreement about the diagnosis, cases were reviewed and adjudicated in conjunction with a third cardiologist. Adjudication of the final diagnosis was performed centrally in a core laboratory and included two sets of serial (hs-)cTn measurements: serial (hs)-cTn measurements obtained as part of routine clinical care locally (different (hs)-cTn assays) and serial measurements of hs-cTnT from study blood draws performed centrally in the core laboratory in order to take advantage of the higher sensitivity and higher overall diagnostic accuracy offered by hs-cTnT [2].

T1MI and T2MI were defined according to the fourth universal definition of MI [2]. In addition to the evidence of myocardial necrosis in a clinical setting consistent with acute myocardial ischemia, T1MI was defined as spontaneous MI related to a primary atherothrombotic coronary event such as plaque erosion or rupture, intraluminal coronary thrombus, or distal microembolization. T2MI was defined as secondary to an oxygen supply–demand mismatch in the context of brady- or tachyarrhythmias, hypoxemia, hypotension, hypertension, severe anemia, or coronary artery spasm, coronary embolism, and non-atherosclerotic dissection. An underlying coronary artery disease was possible, but not required, for T2MI. To qualify for T2MI, the same dynamic changes in hs-cTn were required as for T1MI. As recommended, the documentation of a clear trigger was essential for the diagnosis of T2MI. Coronary angiography was not mandatory for a diagnosis of T1MI to limit the effect of selection bias due to clinical referral to coronary angiography. Also, as indicated in the fourth universal definition of MI, different etiologies of acute cardiomyocyte injury such as myocarditis, takotsubo syndrome, and acute heart failure were adjudicated as other cardiac pathologies, distinct from T1MI and T2MI.

### 2.4. Blood Sampling and Laboratory Methods

Blood samples for the determination of hs-cTn were collected at presentation to the ED and 1 and 2 h after presentation. After centrifugation, samples were frozen at −80 °C until assayed in a blinded fashion in a dedicated core laboratory.

The Roche hs-cTnT assays used the Elecsys 2010 system (Roche Diagnostics, Rotkreuz, Switzerland), with a limit of detection (LoD) of 5 ng/L, a 99th-percentile cut-off point of 14 ng/L, and a coefficient of variation (CV) of less than 10% at 13 ng/L [13,14,15].

The Abbott Architect hs-cTnI assay used was the ARCHITECT High-Sensitivity STAT Troponin I assay (Abbott Laboratories, Abbott Park, IL, USA). The Abbott Architect hs-cTnI assay was performed with the use of the Architect system with a LoD of 1.9 ng/L and a 99th percentile cut-off point of 26.2 ng/L with a corresponding CV of <5% [16].

### 2.5. Calculation of the Neumann Score

The clinical risk score was applied as recommended [10]. In brief, the Neumann Score included female sex, no radiating chest pain, and a baseline hs-cTnI concentration ≤40.8 ng/L and assigned 1 point per variable, obtaining a total score ranging from 0 to 3 points (Supplementary Table S2). The predicted probability of having T2MI is given by

(1) $$p=\;\frac{1}{1+e^{-\left(Intercept+\;Points\right)}}$$

### 2.6. Neumann Risk Model

The Neumann Score was derived from a multivariable logistic model by conversion of the regression coefficients (beta coefficients) to a point-based score. Physicians will use the score for decision-making, therefore performance measurements of the score, and not the risk model, are of main importance. However, for better insight, we provide calibration plots for the risk model, as well as for the point-based score. Calibration plots using beta coefficients for calculating the predicted probability can be found in Supplementary Figures S1 and S2.

The probability of T2MI in individual patients is given by

(2) $$p=\;\frac{1}{1+e^{-\left(\beta_{0}\;\;+\;\beta_{1}\times predictor_{1}\;\;+\;\ldots \;+\;\beta_{n}\times predictor_{n}\right)\prime }}$$

where *p* stands for the predicted probability, $\beta_{0}\;$ for the intercept, and $\beta_{n}$ for the coefficients of the risk variables.

### 2.7. Objective

The primary objective was to evaluate the model performance of the Neumann Score.

### 2.8. Statistical Analysis

Continuous variables are presented as medians (interquartile range (IQR)); categorical variables as numbers and percentages. Differences in baseline characteristics between patients with T1MI and T2MI were assessed using the Mann–Whitney U test for continuous variables and the Pearson Chi-square test for categorical variables. Confidence intervals (CI) of proportions were computed as appropriate [17].

The Neumann Score was externally validated using methods described previously [18,19]. To study the performance of the Neumann Score in our validation cohort, we assessed its discrimination and calibration. Discrimination is the ability of the score or model to discriminate patients with T2MI from patients without T2MI. Score discrimination was quantified with the area under the curve (AUC), which is equal to the concordance statistics (c-statistic) for a dichotomous outcome variable [20]. Calibration refers to the agreement between the predicted risk and the observed frequencies of T2MI. The model’s calibration was graphically assessed through a calibration plot. The calibration plot and its parameters (intercept and slope) were first estimated without adjustment. The score was then recalibrated by adjustment of the intercept (adjustment for baseline risk) [18].

To extend the Neumann Score, multivariable logistic regression analysis was used to determine whether adding different clinical variables to the Neumann Score variables improved model performance. The Neumann Score variables were entered as a score with fixed coefficients rather than as separate variables to prevent the original Neumann Score coefficients to be adjusted in the presence of the new variables, hence modifying the whole risk score. Clinical variables were selected based on feasibility, clinical meaningfulness, and current guidelines. Stepwise selection of additional predictors was used to evaluate potential predictors which were not included in the original model [21]. As the objective was to improve model performance, Brier Score, AUC, and *p* value of the Hosmer–Lemeshow statistic were assessed for deciding the best final model. Once the final model was chosen, the regression coefficients of the new variables were transformed into a point-based score and added to the Neumann Score, resulting in the Extended Neumann Score.

We assessed improvement in model performance by calculating the change in the AUC, the relative integrated discrimination improvement (IDI), the net reclassification improvement (NRI), and the net benefit in the form of decision curve analysis (DCA), as recommended by the TRIPOD statement [11]. Relative IDI expresses the improvement in T2MI predicted probability on a percentage scale for the Neumann Score and the Extended Neumann Score [22]. The NRI quantifies the ability of the Extended Neumann Score to reclassify patients by counting how many patients with T2MI were reclassified to a higher probability category and how many without T2MI (T1MI) were reclassified to a lower probability category in the Score. We present results for the 2 NRI versions, additive and absolute NRI [23]. Net benefit is a measure that compares benefits and harms. Briefly, it is the difference between the proportion of true positives and the proportion of false positives weighted by the odds of the selected threshold. At any given threshold, the model with the highest net benefit is the preferred model. Net benefit was analyzed using decision curve analysis comparing the Neumann Score with the Extended Neumann Score [24]. Confidence Intervals of AUCs and *p*-values for comparison of AUCs were calculated according to DeLong [25]. Calibration of the extended model was also assessed.

As a performance measure, we report the true positive rate (TPR) and the positive predictive value (PPV) for each point score of the Neumann and Extended Neumann Scores. The PPV is numerically equal to a patient’s post-test probability, i.e., the probability of actually having T2MI when diagnosed as such. The TPR (also known as sensitivity) measures the percentage of true T2MI cases correctly detected by the diagnostic test (in this case, the risk score). Therefore, a good diagnostic test has a high PPV, as well as a high TPR. The rule-in performance is visualized by the TPR and the PPV versus the point-based score. The confidence interval for the TPR and PPV was computed using the Wilsons method [26].

We did not perform formal sample size calculations because the cohort study is an ongoing study. Also, there are no generally accepted approaches to estimate the sample size requirements for external validation studies of risk prediction models [11]. Some have suggested having a minimum of 100 events (type 2 MI, in this case) for external validation of clinical prediction rules [27]. Our sample and the number of events far exceeded all approaches for determining samples sizes and, therefore, were expected to provide very robust estimates.

All hypothesis testing was two-tailed, and *p*-values < 0.05 were considered statistically significant. All statistical analyses were performed using STATA, version 15.1 (Stata Corp, College Station, TX, USA), and R, version 3.6.3 (R foundation for Statistical Computing, Free Software Foundation, Boston, MA, USA).

## 3. Results

### 3.1. Patient Characteristics

From April 2006 to April 2018, 6684 patients were recruited. Among them, 1106 (17%) patients with an adjudicated final diagnosis of non- (N) STEMI, 27 (2%) were excluded from this study because they lacked at least one variable for the Neumann Score calculation. A total of 1079 (16%) patients were eligible for the primary analysis. Of these, 242 (22%) had T2MI (Figure 1). The median patient age was 70 years (IQR 59–79). T2MI and T1MI patients were comparable in many baseline characteristics (Table 1). However, T2MI patients were more often female (36% vs. 26%), more likely to present with left bundle branch block (10% vs. 6%) or a higher heart rate (90 vs. 76), less often had previous MI (27% vs. 34%), previous percutaneous coronary intervention (PCI) (24% vs. 32%), and presented with a lower eGFR (69 vs. 75 mL/min/m^2^) and lower systolic blood pressure (134 vs. 145 mmHg). The hs-cTnI concentration at presentation was significantly lower in T2MI compared to T1MI patients (23 vs. 114 ng/L). The most common causes of T2MI (covering 88% of all cases) were arrhythmias, hypertension, anemia, hypoxemia, and coronary spasm (Supplementary Table S3).

### 3.2. External Validation of the Neumann Score

**Figure 2:**
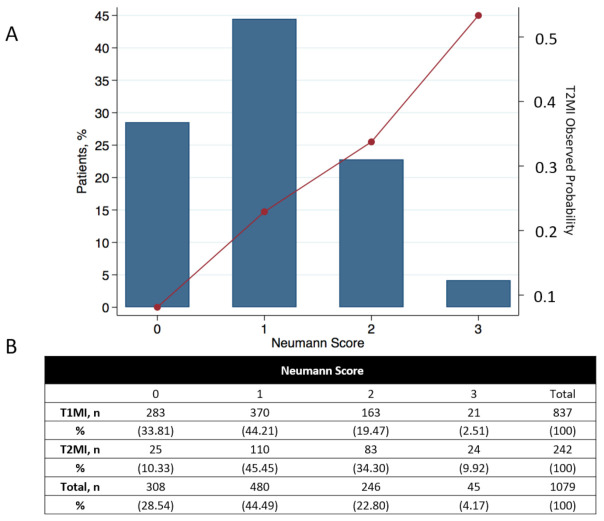
Neumann risk score distribution and relationship with the observed probability of T2MI for each score. (**A**) Distribution of the calculated Neumann risk score and its relationship with the observed probability of T2MI in all 1079 patients. (**B**) Total number of AMI patients for each score and patient stratification for T1MI and T2MI. Percentages show the number of patients in each group with respect to the total number of T1MI or T2MI patients (left Y-axis). For calculating the observed probability of T2MI in the validation cohort, the reader only needs to divide the number of T2MI/all patients for the desired score (e.g., for a 3-point score, the observed probability of T2MI would be 24/45 = 53.3%). The observed probability for each point score appears in the right Y-axis.

**Figure 3:**
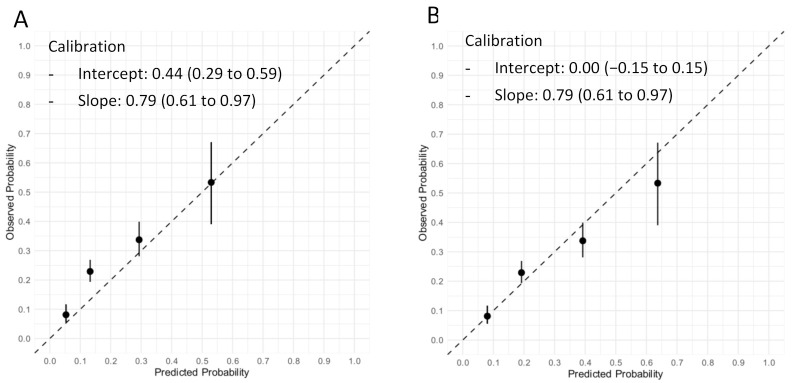
Calibration plot of the original and recalibrated Neumann Score tested in the validation cohort. Assessment of goodness of fit. (**A**) Calibration plot of the Neumann Score tested in the validation cohort. The highest predicted probability (0.53) is obtained with a score of 3. An intercept >0 indicates that the score’s predicted probabilities in the validation cohort are systematically too high. (**B**) Calibration plot of the recalibrated Neumann Score tested in the validation cohort. The highest predicted probability (0.64) is obtained with a score of 3. The agreement between predicted and observed probability appears improved for low risk Scores. An overestimation of the predicted probability can now be observed for the highest score (3). Perfect calibration is represented by the dotted line through the origin. Whiskers indicate 95% CI’s.

The Neumann Score distribution and its relationship with the observed probability of T2MI is shown in Figure 2. The diagnostic accuracy of the Neumann Score was moderate, with a c-statistic of 0.67 (95% CI 0.64–0.71). The calibration plot showed a moderate agreement between the predicted probabilities of T2MI according to the Neumann Score and the observed frequencies (Figure 3A). The predicted probabilities were underestimated for the lowest risk groups (score 0–2) assessed by visual inspection and confirmed with a calibration intercept of 0.44 (CI, 0.29–0.59). An intercept >0 indicates that the score’s predicted probabilities in the validation set are systematically too low. Agreement for the highest risk group (Score of 3) was optimal. The calibration slope was 0.79 (CI, 0.61–0.97). The Hosmer–Lemeshow test for external validation yielded a *p*-value < 0.001, indicating suboptimal agreement.

To adjust for different baseline risks between the derivation cohort and our external validation cohort, the Neumann Score was recalibrated. The recalibrated Neumann Score showed improved calibration for the lower risk groups (risk scores 0–2), with correction of the underestimation for these groups indicated by the calibration points appearing nearer to the optimal line (45° dotted line). However, a modest overestimation for the highest risk group (risk score 3) was now observed (Figure 3B). With regard to the overall calibration, the calibration slope was maintained (0.79 (CI, 0.61–0.97)). After recalibration, the Hosmer–Lemeshow test yielded a *p*-value of 0.20.

The calibration plots of the prediction model using original beta regression coefficients instead of the point-based score can be found in Supplementary Figure S1**.**

### 3.3. Extension of the Neumann Score

**Figure 4:**
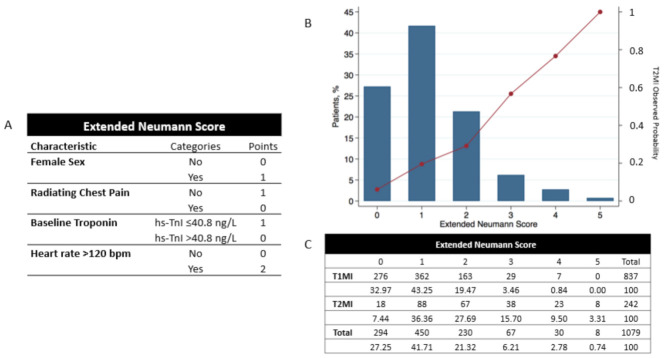
Extended Neumann Score, its distribution, and its relationship with the observed probability of T2MI for each Score. (**A**) Extended Neumann risk score. (**B**) Distribution of the calculated Extended Neumann risk score and its relationship with the observed probability of T2MI for all 1079 patients. (**C**) Total number of AMI patients for each score and stratification for T1 and T2MI. Percentages show the number of patients in each group with respect to the total number of T1 or T2MI patients (left Y-axis). For calculating the observed probability of T2MI in the validation cohort, the reader only needs to divide the number of T2MI/all patients for the desired score (e.g., for a 3-point score, the observed probability of T2MI would be 38/67 = 56.7%). The observed probability for each point score appears in the right Y-axis of (**B**).

Multivariable logistic regression analysis showed that extending the Neumann Score by adding the parameter “*heart rate 120 beats per minute”* significantly improved the diagnostic accuracy assessed by AUC, Brier Score, and Hosmer–Lemeshow test (Table 2). This variable was weighted 2 points if present and 0 points if absent. The Extended Neumann Score, its distribution, and its relationship with the observed frequency of T2MI is shown in Figure 4, central illustration.

**Figure 5:**
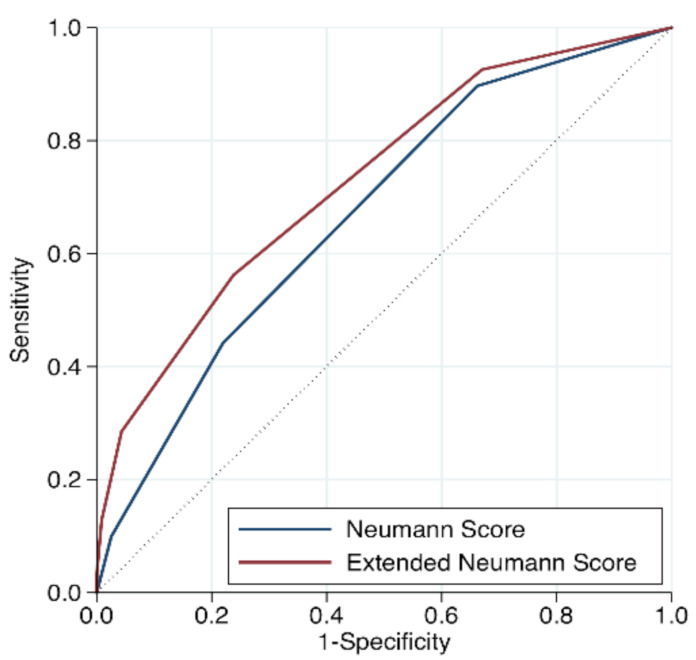
Receiver operating characteristic curve to diagnose type 2 MI for the Neumann and Extended Neumann risk Scores.

Addition of heart rate to the Neumann Score significantly increased the diagnostic accuracy of the Score (c-statistic 0.73; 95% CI 0.70–0.76, *p* < 0.001, Figure 5). Improvement in model performance was also confirmed by improvement in the IDI and NRI. The relative improvement in IDI was 9.7% for predicting T2MI, while the additive NRI was 14.8, and the absolute NRI was 3.2%, favoring the Extended Neumann Score. (Supplementary Table S4). When assessing the additive NRI individually, we obtained better reclassification of patients with T2MI (15.3%) and not worse reclassification of patients without T2MI (incorrect reclassification by 0.1%). In other words, the Extended Neumann Score improved the diagnosis of patients with T2MI without worsening the diagnosis of patients with T1MI.

The decision curve analysis showed improvement in net benefit with the Extended Neumann Score at nearly any threshold probability for diagnosing T2MI (Supplementary Figure S2). The DCA clearly separated from a threshold probability of 0.2 onwards. Given the relative risks of missing a T1MI diagnosis compared to the harms of coronary angiography, we would consider it reasonable for any patient or doctor to demand at least a 70% risk of having T2MI before accepting delayed or no coronary angiography. The net benefit across the high probability threshold range (above 70%) was of approximately 2%. When translated to interventions avoided, the Extended Neumann Score would reduce unnecessary angiographies in comparison to the Neumann Score by an additional 2% for a threshold probability of 70% and by an additional 3% for a threshold probability of 50%.

**Figure 6:**
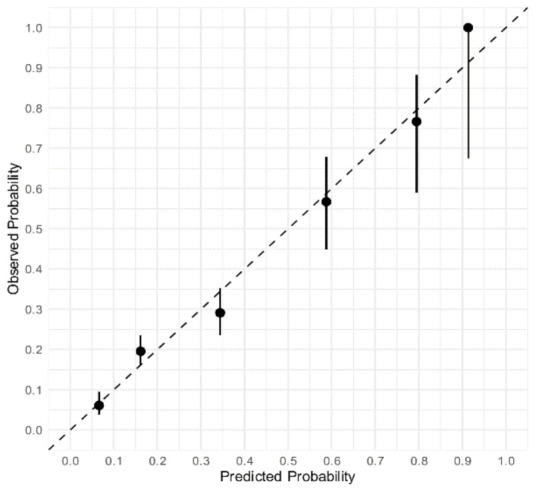
Calibration plot of the extended Neumann Score tested in the validation cohort. Assessment of goodness of fit. Calibration plot of the prediction Score with the extended Neumann risk groups. The highest predicted probability (1.0) is obtained with a score of 5. A nearly optimal calibration slope was achieved. The calibration plot shows good agreement overall, assessed by the proximity of the six scores (0 to 5). Confidence interval increases as sample size reduces in the score groups. Perfect calibration is represented by the dotted line through the origin. Whiskers indicate 95% CIs.

The calibration plot of the Extended Neumann Score showed strong agreement between predicted and observed proportions, obtaining a near optimal slope of 0.91 (CI, 0.75–1.07) and an ideal intercept (0.00 (CI, −0.16 to 0.16), Figure 6). A non-significant Hosmer–Lemeshow test (*p* = 0.51) confirmed the goodness-of-fit. The calibration plots of the prediction model using original beta regression coefficients instead of the point-based score can be found in Supplementary Figure S3.

### 3.4. Diagnostic Performance of the Neumann and Extended Neumann Scores

**Figure 7:**
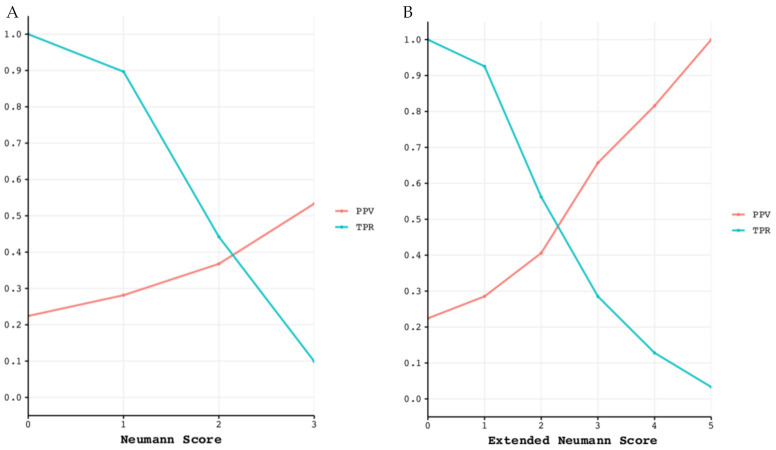
Classification performance for ruling in T2MI. Positive predictive value (PPV) and true positive rate (TPR) for each point of the Neumann (**left**) and Extended Neumann Scores (**right**). (**A**) The maximum reached PPV with the Neuman Score is 53%, meaning that 53% of patients scoring 3 points are correctly diagnosed with T2MI. For a Score of 3, the TPR is 10%, meaning that 10% of all T2MI patients have a score of 3. (**B**) The maximum PPV reached with the Extended Neumann Score is 100%, meaning that all patients scoring 5 points are correctly diagnosed with T2MI. For a score of 5, the TPR is 3.3%, meaning that 3.3% of all T2MI patients have a score of 5.

The diagnostic performance of both risk scores is displayed in Supplementary Table S5. Classification performance for ruling in T2MI is shown in Figure 7.

## 4. Discussion

This large multicenter diagnostic study aimed to contribute to overcoming the clinical uncertainty about the diagnosis T2MI and T1MI by externally validating and extending the recently derived Neumann Score [10]. We report six major findings:

First, in this validation cohort, a Neumann Score (female sex, absence of radiating chest pain, and hs-cTnI-Architect <40.8 ng/L) of 3 points (the highest achievable score) was associated with a 53% observed probability of having T2MI, with only 10% of T2MI patients reaching a score of 3 (Figure 2). Second, the Neumann Score showed moderate discrimination (AUC 0.67, 95%CI 0.64–0.71), comparable to that observed in the Hamburg single-center derivation cohort (AUC 0.71, 95%CI 0.67–0.79) [10]. Third, the calibration plot showed a positive intercept, which indicated an overall underestimation for predicting T2MI risk. Fourth, extending the Neumann Score by adding heart rate at presentation >120/min as an additional binary variable increased the diagnostic accuracy to 0.73 (95%CI 0.70–0.76, *p* < 0.001). Improvement in model performance was also confirmed by improvement in IDI, NRI, and DCA. Fifth, the Extended Neumann Score showed good overall calibration (Hosmer–Lemeshow test, *p* = 0.51). A Visual examination of the calibration plot demonstrated strong agreement between the predicted probabilities and the observed proportions of T2MI along all score groups. Sixth, 5 points (highest score) for the Extended Neumann Score corresponded to 100% observed probability of T2MI, although a minority of patients reached it (3%). When a score is derived with the aim to diagnose a disease which has primarily a non-invasive therapy (T2MI) in contrast to other possible diagnoses which should receive an invasive procedure (coronary angiography, T1MI), the score should be favored for a very high PPV (high safety) and reduce the number of false positives (false positive = the patient has T1MI but is classified as a T2MI patient by the score and does not receive coronary angiography). Therefore, the Extended Neumann Score represents a clinically meaningful improvement versus the original score [10]. However, due to the only moderate performance of both scores, their clinical implementation may be of limited usefulness for the clinician. Further improvement is necessary, possibly by adding additional cardiovascular biomarkers and other criteria. Additionally, the evaluation of a score to predict short- and long-term outcomes in T2MI and T1MI patients is a major clinical need for further improvement of care, especially for T2MI.

Still, the low percentage of correctly classified T2MI patients indicates that in many patients, invasive or non-invasive anatomical and/or functional cardiac imaging including coronary angiography will remain necessary for the accurate differentiation of T2MI with respect to T1MI [2]. Particularly when deriving and validating prediction rules for T2MI, it is mandatory to strictly adhere to the universal definition of MI [2] and not lump patients with cardiomyocyte injury of unknown or miscellaneous cause such as acute heart failure, Takotsubo syndrome, and myocarditis also into the group of T2MI. This error could overestimate the predicted probability of having T2MI.

Several limitations should be considered when interpreting these findings. First, this study was conducted in patients presenting to the ED. We cannot comment on the performance of the Extended Neumann Score in other clinical settings including the perioperative setting and critically ill patients. Second, although we used a very stringent methodology to adjudicate T1MI and T2MI including central adjudication by experienced cardiologists using cardiac imaging and serial measurements of hs-cTn, we may still have misclassified a small number of patients [2]. This could have led to an underestimation of the true accuracy of the Neumann Score and the Extended Neumann Score. Third, the Neumann Score includes hs-cTnI measured with one specific analyzer. Future studies need to define the optimal hs-cTnI/T cut-offs for other clinically used hs-cTnI/T assays. Fourth, we cannot generalize these findings to patients with terminal kidney failure requiring dialysis, since these patients were excluded from this study.

## 5. Conclusions

In conclusion, external validation of the Neumann Score showed moderate discrimination and suboptimal calibration. Extending the Neumann Score by adding the parameter heart rate >120/min significantly improved the model’s performance.

## Figures and Tables

**Table 1:** Baseline Characteristics in patients with AMI, T1MI and T2MI.

Baseline Characteristics	All AMI Patients (*n* = 1079)	T1MI (*n* = 837)	T2MI (*n* = 242)	*p* Value
Age, year	70.0 (59.0, 79.0)	70.0 (58.0, 79.0)	72.0 (60.0, 80.0)	0.33
Female	307 (28.5%)	220 (26.3%)	87 (36.0%)	0.003
Cardiovascular risk factors, *n* (%)				
Hypertension	832 (77.1%)	645 (77.1%)	187 (77.3%)	0.94
Hypercholesterolemia	671 (62.2%)	543 (64.9%)	128 (52.9%)	<0.001
Diabetes mellitus	288 (26.7%)	225 (26.9%)	63 (26.0%)	0.79
Current smoker	251 (23.3%)	207 (24.7%)	44 (18.2%)	0.034
Former smoker	438 (40.6%)	343 (41.0%)	95 (39.3%)	0.63
History, *n* (%)				
Coronary artery disease	480 (44.5%)	380 (45.4%)	100 (41.3%)	0.26
Previous MI	353 (32.7%)	287 (34.3%)	66 (27.3%)	0.04
Previous CABG	160 (14.8%)	128 (15.3%)	32 (13.2%)	0.42
Previous PCI	329 (30.5%)	270 (32.3%)	59 (24.4%)	0.019
Peripheral artery disease	122 (11.3%)	98 (11.7%)	24 (9.9%)	0.44
Previous stroke	97 (9.0%)	78 (9.3%)	19 (7.9%)	0.48
ECG findings, *n* (%)				
Left bundle-branch block	73 (6.8%)	49 (5.9%)	24 (9.9%)	0.027
ST-segment depression	111 (10.3%)	79 (9.4%)	32 (13.2%)	0.088
T-wave inversion	243 (22.5%)	196 (23.4%)	47 (19.4%)	0.19
Laboratory findings				
hs-TnI on admission (ng/L)	76.3 (20.3, 423.8)	114.0 (28.0, 576.1)	23.1 (10.0, 83.1)	<0.001
Haemoglobin (g/dL)	141.0 (127.0, 153.0)	142.0 (127.0, 153.0)	140.0 (125.0, 152.0)	0.15
eGFR, (mLmin/m^2^)	73.6 (57.8, 92.2)	75.3 (60.0, 94.1)	69.2 (52.1, 88.6)	<0.001
Vital signs on admission				
Systolic blood pressure (mmHg)	141.0 (125.0, 160.0)	145.0 (128.0, 161.0)	134.0 (116.0, 153.0)	<0.001
Heart rate (bpm)	78.0 (68.0, 92.0)	76.0 (66.0, 88.0)	89.5 (73.0, 120.0)	<0.001
Respiratory rate (per minute)	16.0 (14.0, 20.0)	16.0 (14.0, 20.0)	16.0 (14.0, 20.0)	0.76
SaO2 (%)	98.0 (96.0, 99.0)	98.0 (96.0, 99.0)	98.0 (96.0, 99.0)	0.71
Chest pain characteristics				
No radiating chest pain	388 (36.0%)	280 (33.5%)	108 (44.6%)	0.001
Admission medication				
Antiaggregant (aspirin + Clopidogrel)	549 (50.9%)	440 (52.6%)	109 (45.0%)	0.039
Warfarin (Vitamin K antagonist)	125 (11.6%)	81 (9.7%)	44 (18.2%)	<0.001
Beta-blocker	460 (42.6%)	342 (40.9%)	118 (48.8%)	0.029
Statin	481 (44.6%)	375 (44.8%)	106 (43.8%)	0.78
ACEIs/ARBs	574 (53.2%)	443 (52.9%)	131 (54.1%)	0.74
Calcium antagonists	220 (20.4%)	174 (20.8%)	46 (19.0%)	0.54
Nitrates	165 (15.3%)	139 (16.6%)	26 (10.7%)	0.026

Table Legend: Displayed are the baseline characteristics for all AMI patients and stratification by AMI type. The *p*-value is given for the comparison between T1MI and T2MI patients. MI, myocardial infarction; T1MI, type 1 myocardial infarction; T2MI, type 2 myocardial infarction; CABG, coronary artery bypass graft; PCI, percutaneous coronary intervention; eGFR, estimated glomerular filtration rate; SaO2, oxygen saturation; ACEI/ARB, angiotensin converting enzyme inhibitor/angiotensin receptor blocker, ECG, electrocardiogram.

**Table 2:** Multivariable logistic regression analysis for the diagnosis of T2MI.

	Multivariable Analysis					
Variables	Beta Coefficient (95% CI)	OR (95% CI)	*p*-Value	Brier	AUC	hl
Age < 70 years	0.119 (−0.187–0.423)	1.126 (0.829–1.526)	0.446	0.163	0.671	0.138
Heart rate > 120/min	2.472 (1.935–3.038)	11.842 (6.927–20.866)	<0.001	0.145	0.732	0.239
Systolic blood pressure > 160 mmHg	−0.350 (−0.730–0.016)	0.705 (0.482–1.017)	0.066	0.163	0.68	0.142
No previous coronary artery disease	0.218 (−0.088–0.527)	1.244 (0.915–1.694)	0.165	0.162	0.673	0.047
No previous myocardial infarction	0.434 (0.102–0.774)	1.543 (1.108–2.167)	0.011	0.161	0.679	0.038
No pathological ECG changes	−0.155 (−0.459–0.149)	0.857 (0.632–1.161)	0.318	0.163	0.675	0.137
eGFR ≤ 30 mL/min/1.73 m^2^	0.557 (−0.133–1.214)	1.746 (0.876–3.365)	0.103	0.163	0.677	0.263
Heart Rate > 120/min + no previous MI				0.145	0.736	0.066

Table Legend: Provided is the multivariable logistic regression for the diagnosis of T2MI. Female sex, no radiating chest pain, and baseline hs-TnI were fixed in the regression. The predictors were tested one by one. As the objective was to improve model performance, Brier Score, AUC (the area under the curve), and *p* value of the Hosmer–Lemeshow statistics were assessed for deciding the best final model. Heart rate > 120 bpm showed the highest model performance. No improvement to either the area under the curve or the Brier Score was observed by including any additional variables. No improvement to either AUC or the Brier Score was observed when adding the parameter “no previous myocardial infarction” to “heart rate > 120 beats per minute”, while the model’s goodness of fit decreased (*p* value for Hosmer–Lemeshow test = 0.066). Thus, only the predictor “heart rate > 120 beats per minute” was introduced in the Neumann Score; OR, odds ratio; hl: Hosmer–Lemeshow; CI, confidence interval; ECG, electrocardiogram.

## Data Availability

Data is available upon request to the corresponding author.

## Supplementary Materials

The following are available online at https://www.mdpi.com/2077-0383/10/6/1264/s1, Figure S1A: Calibration plot of the Neumann prediction model, Figure S1B: Calibration plot of the recalibrated Neumann prediction model, Figure S2: Decision curve analysis of net benefit for heart rate addition (Extended Neumann Score) to the Neumann Score for diagnosis of T2MI, Figure S3: Calibration plot of the Extended Neumann prediction model, Table S1: TRIPOD checklist, Table S2: Original Neumann Score, Table S3: T2MI underlying main triggers, Table S4: Risk reclassification for diagnosis of T2MI based on the addition of heart rate (Extended Score) to the Neumann Score, Table S5A: Diagnostic performance of the Neumann Score, Table S5B: Diagnostic performance of the Extended Neumann Score.
