# Supplementary material for: External Validation and Extension of a Clinical Score for the Discrimination of Type 2 Myocardial Infarction
Source: J Clin Med. 2021 Mar 18;10(6):1264. doi: 10.3390/jcm10061264 (PMC8003225; doi:10.3390/jcm10061264)
Supplement: Supplementary file 1 [file jcm-10-01264-s001.pdf]

External Validation and Extension of a Clinical Score for the Discrimination of Type 2 Myocardial Infarction

Supplementary Material

Supplementary Tables

Supplementary Table S1 – TRIPOD checklist

Supplementary Table S2 – Original Neumann Score

Supplementary Table S3 – T2MI underlying main triggers

Supplementary Table S4 – Risk reclassification for diagnosis of T2MI based on the addition of heart rate (Extended Score) to the Neumann Score

Supplementary Table S5A – Diagnostic performance of the Neumann Score

Supplementary Table S5B – Diagnostic performance of the Extended Neumann Score

Supplementary Figures

Supplementary Figure S1A – Calibration plot of the Neumann prediction model

Supplementary Figure S1B – Calibration plot of the recalibrated Neumann prediction model

Supplementary Figure S2 – Decision curve analysis of net benefit for heart rate addition (Extended Neumann Score) to the Neumann Score for diagnosis of T2MI

Supplementary Figure S3 – Calibration plot of the Extended Neumann prediction model

Supplementary Table S1 TRIPOD Checklist

Section/Topic			Checklist Item	Page
**Title and abstract**				
Title	1	D;V	Identify the study as developing and/or validating a multivariable prediction model, the target population, and the outcome to be predicted.	1
Abstract	2	D;V	Provide a summary of objectives, study design, setting, participants, sample size, predictors, outcome, statistical analysis, results, and conclusions.	2
**Introduction**				
Background and objectives	3a	D;V	Explain the medical context (including whether diagnostic or prognostic) and rationale for developing or validating the multivariable prediction model, including references to existing models.	5
	3b	D;V	Specify the objectives, including whether the study describes the development or validation of the model or both.	5
**Methods**				
Source of data	4a	D;V	Describe the study design or source of data (e.g., randomized trial, cohort, or registry data), separately for the development and validation data sets, if applicable.	6
	4b	D;V	Specify the key study dates, including start of accrual; end of accrual; and, if applicable, end of follow-up.	6
Participants	5a	D;V	Specify key elements of the study setting (e.g., primary care, secondary care, general population) including number and location of centres.	6
	5b	D;V	Describe eligibility criteria for participants.	6
	5c	D;V	Give details of treatments received, if relevant.	n/a
Outcome	6a	D;V	Clearly define the outcome that is predicted by the prediction model, including how and when assessed.	7-9
	6b	D;V	Report any actions to blind assessment of the outcome to be predicted.	n/a
Predictors	7a	D;V	Clearly define all predictors used in developing the multivariable prediction model, including how and when they were measured.	7-9
	7b	D;V	Report any actions to blind assessment of predictors for the outcome and other predictors.	n/a
Sample size	8	D;V	Explain how the study size was arrived at.	13
Missing data	9	D;V	Describe how missing data were handled (e.g., complete-case analysis, single imputation, multiple imputation) with details of any imputation method.	9
Statistical analysis methods	10a	D	Describe how predictors were handled in the analyses.	n/a
	10b	D	Specify type of model, all model-building procedures (including any predictor selection), and method for internal validation.	n/a
	10c	V	For validation, describe how the predictions were calculated.	9-12
	10d	D;V	Specify all measures used to assess model performance and, if relevant, to compare multiple models.	9-12
	10e	V	Describe any model updating (e.g., recalibration) arising from the validation, if done.	9-12
Risk groups	11	D;V	Provide details on how risk groups were created, if done.	n/a
Development vs. validation	12	V	For validation, identify any differences from the development data in setting, eligibility criteria, outcome, and predictors.	9-12
**Results**				
Participants	13a	D;V	Describe the flow of participants through the study, including the number of participants with and without the outcome and, if applicable, a summary of the follow-up time. A diagram may be helpful.	13, Suppleme ntary Fig 1
	13b	D;V	Describe the characteristics of the participants (basic demographics, clinical features, available predictors), including the number of participants with missing data for predictors and outcome.	13, (Table 1)
	13c	V	For validation, show a comparison with the development data of the distribution of important variables (demographics, predictors and outcome).	Suppleme ntary Table 3
Model development	14a	D	Specify the number of participants and outcome events in each analysis.	n/a
	14b	D	If done, report the unadjusted association between each candidate predictor and outcome.	n/a
Model specification	15a	D	Present the full prediction model to allow predictions for individuals (i.e., all regression coefficients, and model intercept or baseline survival at a given time point).	n/a
	15b	D	Explain how to use the prediction model.	n/a
Model performance	16	D;V	Report performance measures (with CIs) for the prediction model.	13-14 (Figure 2)
Model-updating	17	V	If done, report the results from any model updating (i.e., model specification, model performance).	14-15 (Fig. 4-6) (Table 2)
**Discussion**				
Limitations	18	D;V	Discuss any limitations of the study (such as nonrepresentative sample, few events per predictor, missing data).	17
Interpretation	19a	V	For validation, discuss the results with reference to performance in the development data, and any other validation data.	16-18
	19b	D;V	Give an overall interpretation of the results, considering objectives, limitations, results from similar studies, and other relevant evidence.	16-18
Implications	20	D;V	Discuss the potential clinical use of the model and implications for future research.	16-18
**Other information**				
Supplementary information	21	D;V	Provide information about the availability of supplementary resources, such as study protocol, Web calculator, and data sets.	6
Funding	22	D;V	Give the source of funding and the role of the funders for the present study.	21

D stands for derivation; V stands for validation

**Supplementary Table S2 Original Neumann Score**		
**Characteristic**	Categories	Points
**Female Sex**	No	0
	Yes	1
**Radiating Chest Pain**	No	1
	Yes	0
**Baseline Troponin**	hs-TnI ≤40.8 ng/L	1
	hs-TnI >40.8 ng/L	0

Hs-TnI denotes high-sensitivity Troponin I

**Supplementary Table S3 T2MI main triggers**		
Trigger of T2MI	n	%
Tachycardia	134	55.4
Bradycardia	10	4.1
Hypertension	44	18.2
Hypotension	5	2.1
Anaemia	13	5.4
Hypoxaemia	12	5
Coronary dissection	1	0.4
Vasopasm	13	5.4
Embolism	1	4
Total	233	96.3
Others	9	3.7
Total	242	100

In 9 patients a single trigger was not clear, hence classified as Others.

T2MI denotes type 2 myocardial infarction

Supplementary Table S4 Risk reclassification for diagnosis of T2MI based on the addition of heart rate (Extended Score) to the Neumann Score

Neumann Score	Neumann Score + heart rate >120 bpm (Extended Score)							
	Patients with T2MI (n=242)				Patients without T2MI (n=837)			
	<10%	10-30%	30-50%	≥50%	<10%	10-30%	30-50%	≥50%
<10%	**18**	0	0	7	**276**	0	0	7
10-30%	0	**97**	0	24	0	**405**	0	11
30-50 %	0	0	**51**	21	0	0	**113**	4
≥50%	0	0	16	**8**	0	0	21	**0**

	Patients with T2MI, No	Patients without T2MI, No		
Correct reclassification	52	21	Additive NRI	14.8
Incorrect reclassification	16	22	Absolute NRI	3.2%
Net reclassification	36	-1		

T2MI denotes type 2 myocardial infarction; NRI denotes net reclassification index.

Supplementary Table S5A Diagnostic performance of the Neumann point-based Score

Neumann Score	Sensitivity	Specificity	PPV	NPV	TP	FP	TN	FN	Total number of T2MI cases	Total number of AMI cases
0	100 (98.4 - 100)	0 (0.0 - 0.46)	22.4 (20.0 - 25.0)	NA	242	837	0	0	242	1079
1	89.7 (85.2 - 92.9)	33.8 (30.7 - 37.1)	28.1 (25.1 - 31.4)	91.9 (88.3 - 94.4)	217	554	283	25	242	1079
2	44.2 (38.1 - 50.5)	78.0 (75.1 - 80.7)	36.8 (31.4 - 42.4)	82.9 (80.1 - 85.3)	107	184	653	135	242	1079
3	9.9 (6.8 - 4.3)	97.5 (96.2 - 98.4)	53.3 (39.1 - 67.1)	78.9 (76.3 - 81.3)	24	21	816	218	242	1079

Supplementary Table S5B Diagnostic performance of the Extended Neumann point-based Score

Extended Score	Sensitivity	Specificity	PPV	NPV	TP	FP	TN	FN	Total number of T2MI cases	Total number of AMI cases
0	100 (98.4 - 100)	0 (0.0 - 0.46)	22.4 (20.0 - 25.0)	NA	242	837	0	0	242	1079
1	92.6 (88.6 - 95.2)	33.0 (29.9 - 36.2)	28.5 (25.5 - 31.8)	93.9 (90.5 - 96.1)	224	561	276	18	242	1079
2	56.2 (49.9 - 62.3)	76.2 (73.2 - 79.0)	40.6 (35.5 - 45.9)	85.8 (83.1 - 88.1)	136	199	638	106	242	1079
3	28.5 (23.2 - 34.5)	95.7 (94.1 - 96.9)	65.7 (56.2 - 74.1)	82.2 (79.7 - 84.5)	69	36	801	173	242	1079
4	12.8 (9.2 - 17.6)	99.2 (98.3 - 99.6)	81.6 (66.6 - 90.8)	79.7 (77.2 - 82.1)	31	7	830	211	242	1079
5	3.3 (1.7 - 6.4)	100 (99.5 - 100)	100 (67.6 - 100)	78.2 (75.6 - 80.5)	8	0	837	234	242	1079

PPV denotes positive predictive value; NPV denotes negative predictive value; TP denotes True positive; FP denotes false positive; TN denotes true negative; FN denotes false negative; T2MI denotes type 2 myocardial infarction; AMI denotes acute myocardial infarction

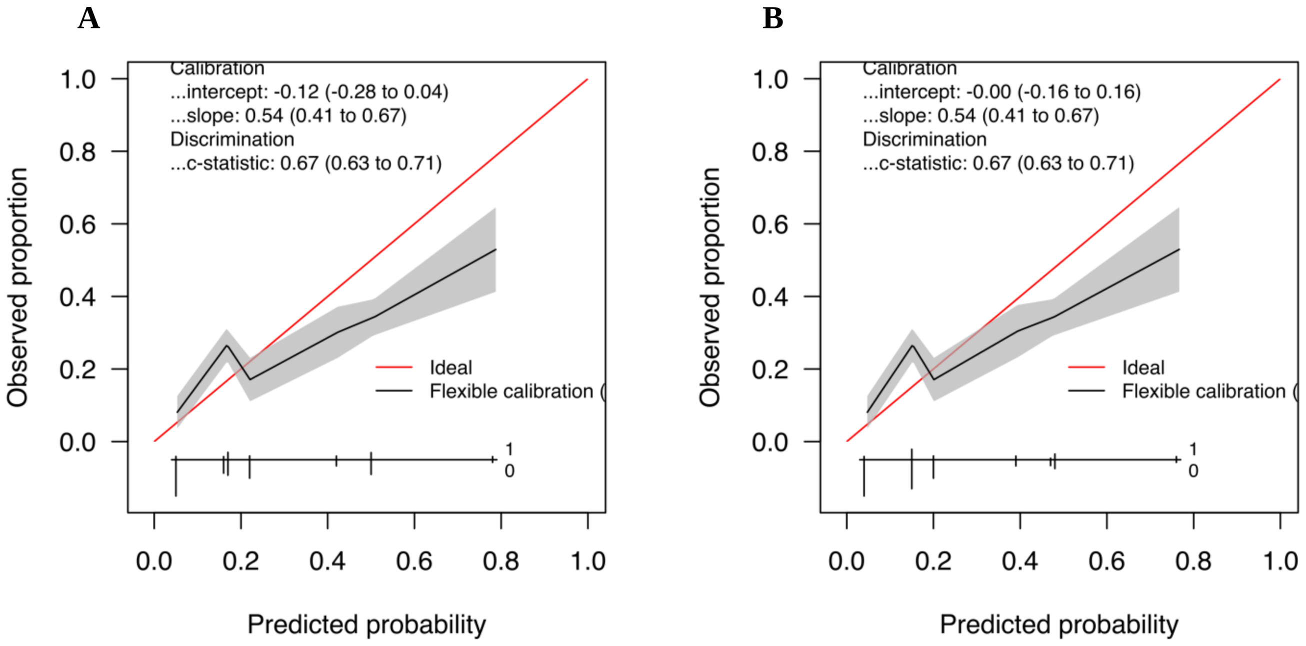

Supplementary Figure S1	Calibration plot of the original and recalibrated prediction model

(A) Calibration plot of the Neumann prediction model (B) Calibration plot of the recalibrated Neumann prediction model. Recalibration did not have a big effect (highest predicted value decreased from 0.79 to 0.76). Perfect calibration is represented by the dotted line through the origin.

The formula of the prediction model is of the form:

$$\log\left(\frac{risk\ of\ T2MI}{1-risk\ of\ T2MI}\right) = \text{linear predictor}$$
$$= \beta_0 + \beta_1 \text{ x predictor}_1 + \ldots + \beta_n \text{ x predictor}_n$$

In this formula, β_0 is the intercept and β_1 till β_n are the regression coefficients, which are presented in the manuscript (**Table 2**).

The risk of T2MI in individual patients can be calculated with the formula:

$$Risk = \frac{1}{1 + e^{-(linear\ predictor)}}$$

The prediction model was transformed into a point-based score (Neumann Risk Score) and one point given to each variable. In the original manuscript (Supplementary material) the calibration plot shown corresponded to the prediction model and not the risk score (hence appearing 8 instead of 4 points). However, a calibration plot of the risk score should have been provided. The authors presented the model as an easy to use risk score chart and this is what physicians will use, hence we have shown in the main manuscript the calibration plot of the risk score, as this will be applied and for taking decisions. Nevertheless, we present also calibration plots from the original and extended prediction model for the interested reader.

Noteworthy, when examining the calibration plot of the prediction model (using beta coefficients), visual inspection shows that the predicted probabilities are overestimated. This is verified by an intercept <0 indicating that the model's predicted probabilities in the validation set are systematically too high. A calibration slope <1 indicates optimism. The beta regression coefficients in the derivation model were too high, resulting in too extreme predictions in new patients (external validation cohort). However, the overestimation is lower in the risk score than in the prediction model. This is due to how the beta-coefficients were transformed into a point-based score. Beta coefficients were rounded down to 1, even if the beta coefficient was higher than 1.5, hence indirectly "correcting" the overfitting (shrinkage of coefficients).

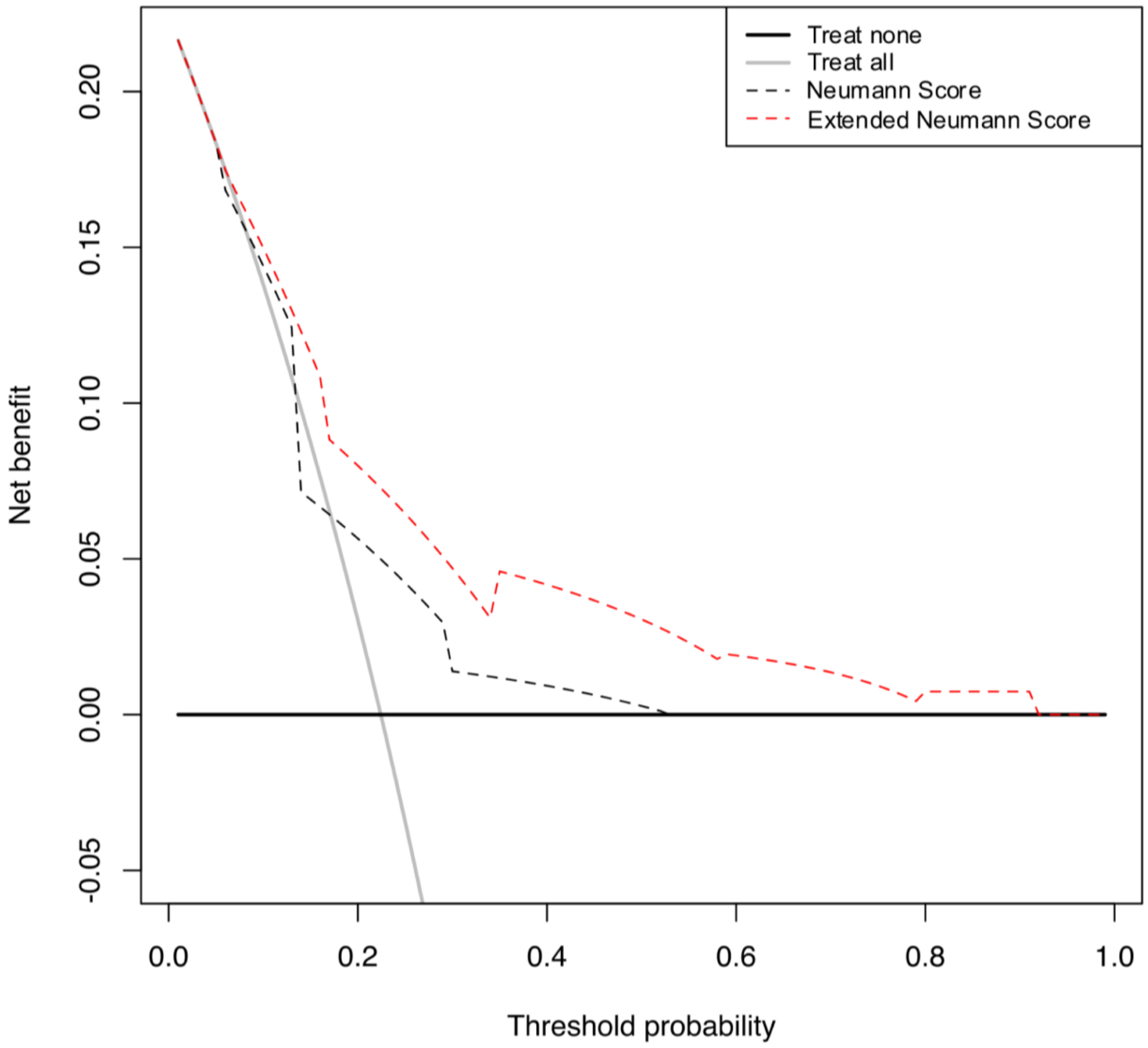

Supplementary Figure S2 **Decision Curve Analysis of Net Benefit for heart rate addition (Extended Neumann Score) to the Neumann Score for diagnosis of T2MI**

The probability threshold captures the relative value the patient/physician places on receiving/providing treatment for the disease (T1MI), if present, to the value of avoiding treatment (angiography) if the disease is not present (T1MI not present). E.g. in a young patient threshold probability will be nearly 1 (higher value on providing treatment for T1MI if present than in avoiding angiography if T1MI not present) obtaining little net benefit with the extended score, while in an older patient, avoiding angiography may be given more relative value than providing treatment for T1MI, if present (e.g. threshold probability of 0.6), hence obtaining more net benefit with the extended score than with the original Neumann Score.

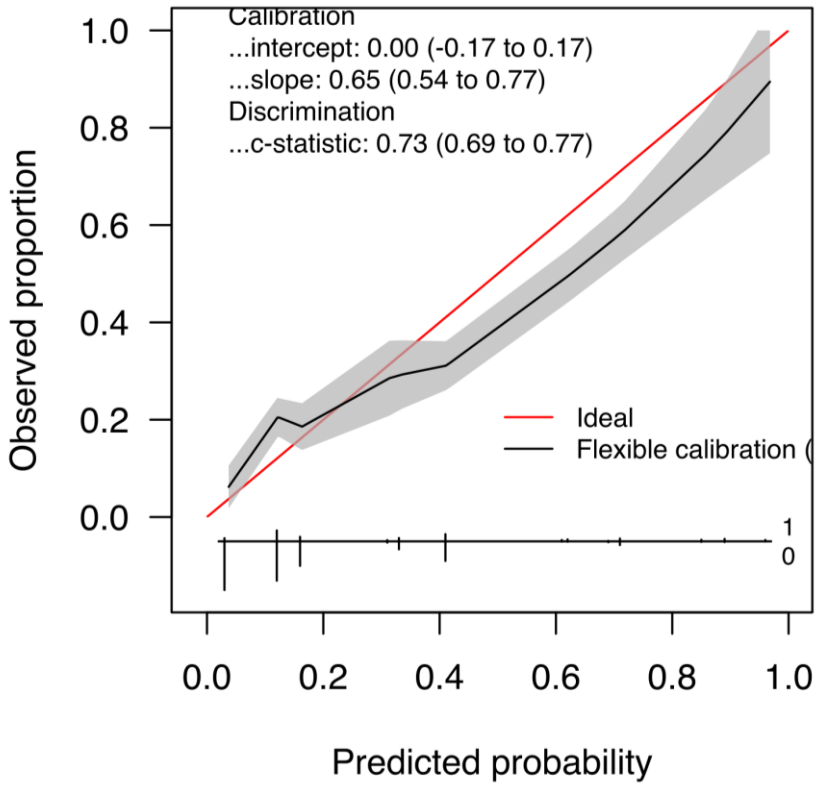

**Supplementary Figure S3**	**Calibration plot of the extended prediction model**

Assessment of goodness of fit. Calibration plot of the extended Neumann prediction model with the beta coefficients. Perfect calibration is represented by the dotted line through the origin.
